# Correction to: SVJedi: genotyping structural variations with long reads

**DOI:** 10.1093/bioinformatics/btad129

**Published:** 2023-03-24

This is a correction notice for: Lolita Lecompte, Pierre Peterlongo, Dominique Lavenier, Claire Lemaitre, SVJedi: genotyping structural variations with long reads, *Bioinformatics*, Volume 36, Issue 17, 1 September 2020, Pages 4568–4575, https://doi.org/10.1093/bioinformatics/btaa527

In the originally published version of this manuscript, there were errors in Figure 5. The metrics reported for the tool svviz2 (in orange color) are identical between the four barplots but should be different. The values are correct for the top left barplot, but incorrect for the other three.

Figure 5 should read:
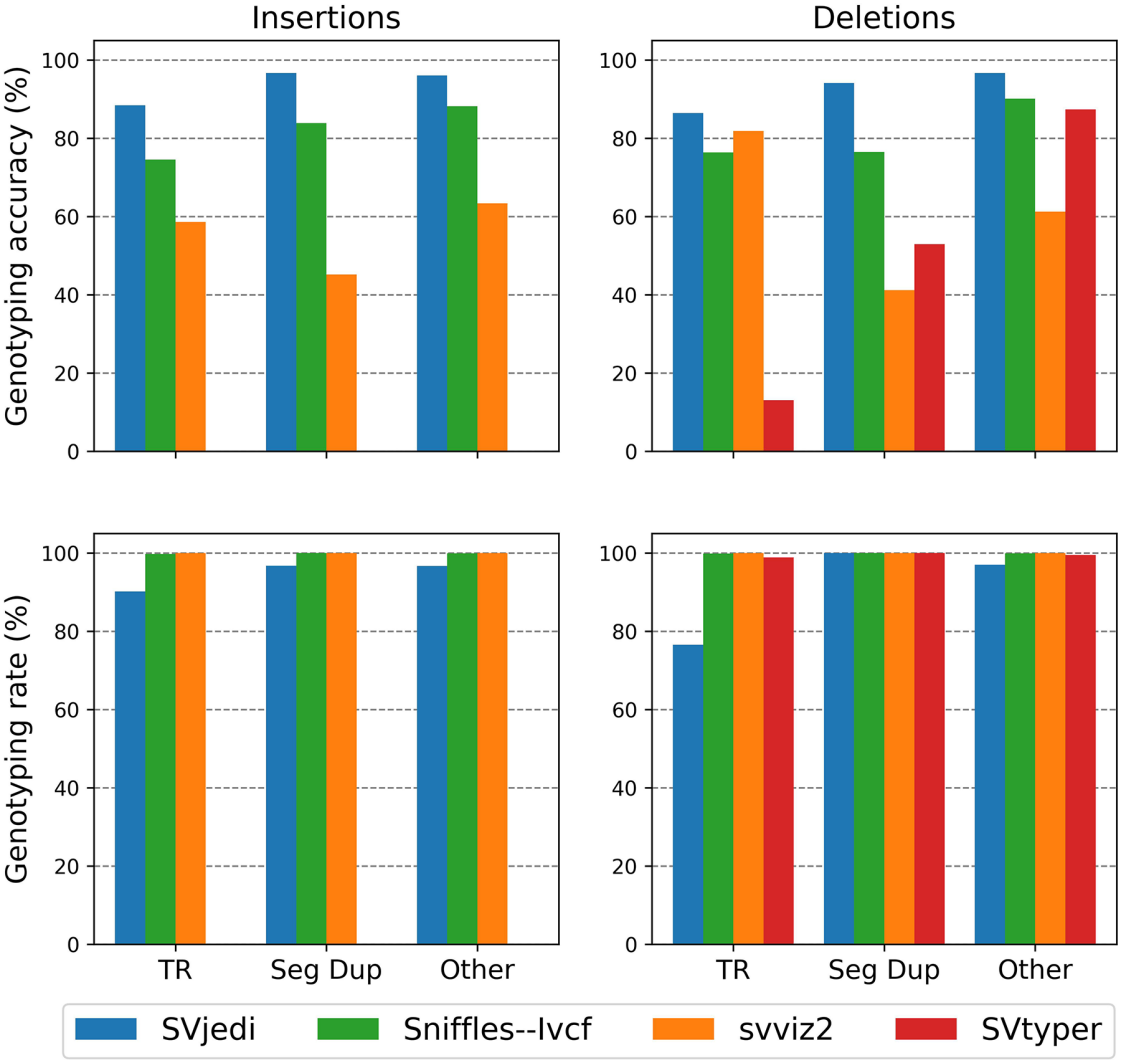


Instead of:
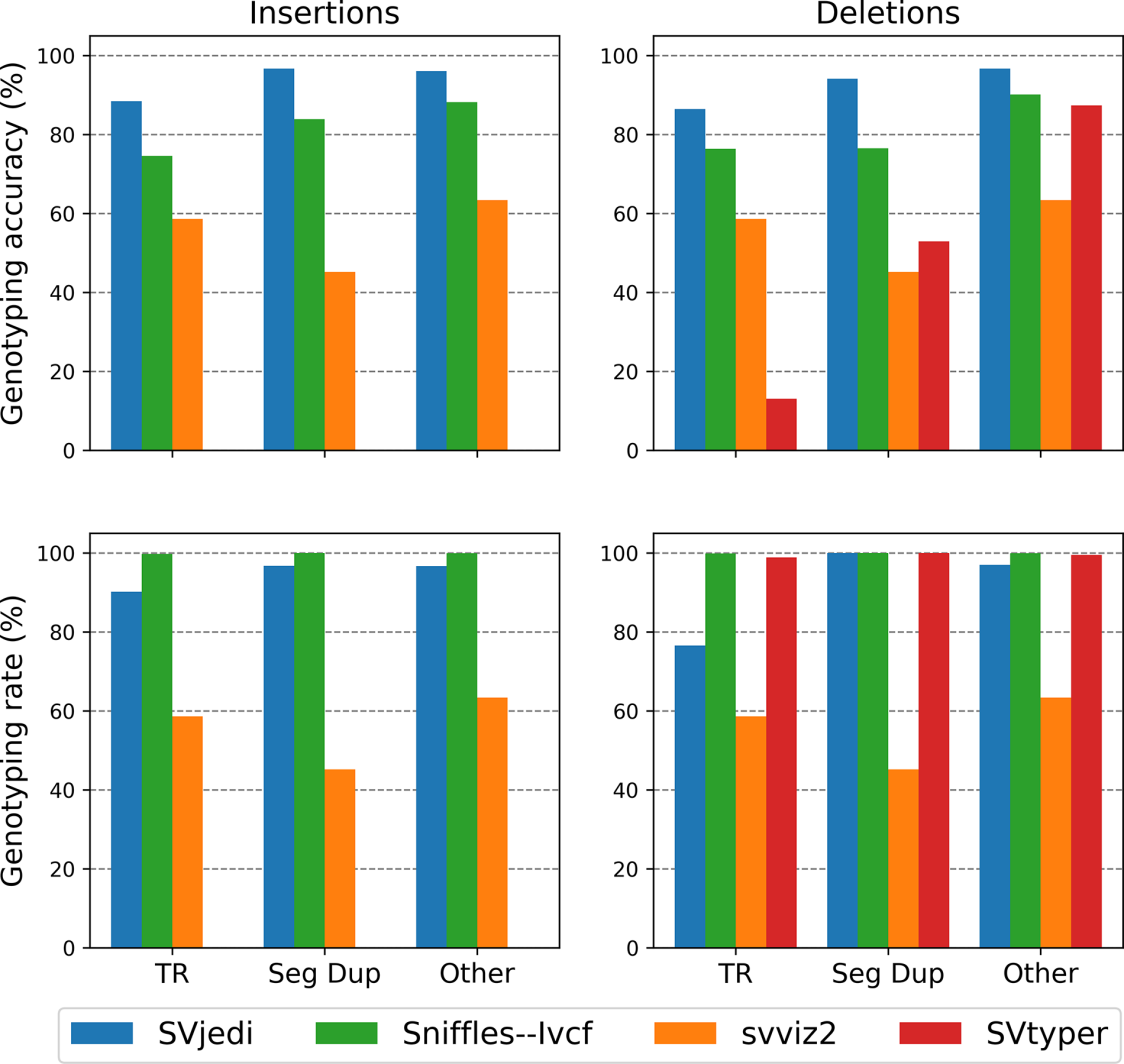


The details have been emended only in correction notice to preserve the published version of record.

